# CFM-ID: a web server for annotation, spectrum prediction and metabolite identification from tandem mass spectra

**DOI:** 10.1093/nar/gku436

**Published:** 2014-06-03

**Authors:** Felicity Allen, Allison Pon, Michael Wilson, Russ Greiner, David Wishart

**Affiliations:** Department of Computing Science, Athabasca Hall, University of Alberta, Edmonton T6G 2E8, Canada.

## Abstract

CFM-ID is a web server supporting three tasks associated with the interpretation of tandem mass spectra (MS/MS) for the purpose of automated metabolite identification: annotation of the peaks in a spectrum for a known chemical structure; prediction of spectra for a given chemical structure and putative metabolite identification—a predicted ranking of possible candidate structures for a target spectrum. The algorithms used for these tasks are based on Competitive Fragmentation Modeling (CFM), a recently introduced probabilistic generative model for the MS/MS fragmentation process that uses machine learning techniques to learn its parameters from data. These algorithms have been extensively tested on multiple datasets and have been shown to out-perform existing methods such as MetFrag and FingerId. This web server provides a simple interface for using these algorithms and a graphical display of the resulting annotations, spectra and structures. CFM-ID is made freely available at http://cfmid.wishartlab.com.

## INTRODUCTION

Metabolomics is a field of omics science that characterizes metabolites using high throughput technologies. Metabolites are all the low molecular weight (<1500 Da) chemicals found in cells, tissues and biofluids (1,2). Electrospray tandem mass spectrometry (ESI-MS/MS) is a widely used technique in untargeted metabolomics experiments (3–6). Manual interpretation of MS/MS spectra to aid metabolite identification is known to be both time-consuming and tedious. Automating this process promises to offer substantial time and cost benefits.

To this end, we have released CFM-ID, a web server that provides three utilities that address important subtasks of the metabolite identification problem: MS/MS spectrum prediction, MS/MS peak annotation and putative metabolite identification (7). These utilities, which will be further described below, present web-based front-ends to the functionality provided by the Single Energy Competitive Fragmentation Modeling (SE-CFM) technique introduced in (8).

We envision that this functionality could be particularly useful to experimentalists, as it will help them perform some of the more time-consuming tasks in the interpretation of mass spectrometry data. For example, one possible use is to help analyze an MS/MS spectrum for an unknown compound. Users would first produce a list of candidate molecules, e.g. by querying a public chemical repository for molecules of the correct mass. They might also be able to refine that list by incorporating other analytical information (e.g. information from NMR spectra), or knowledge about where the molecule was found (e.g. in human blood serum). Once they have a suitable list, they could then use our Compound Identification tool to rank those candidates to produce a shorter list. They might also use our Spectrum Prediction tool to examine the predicted spectrum for each of those candidates to manually verify the match, or use our Peak Assignment tool to investigate how the peaks in the measured spectrum could have formed from each candidate. This might help them to direct their subsequent experimentation to further verify the identity of the metabolite of interest.

## WEB SERVER

**Figure 1.**
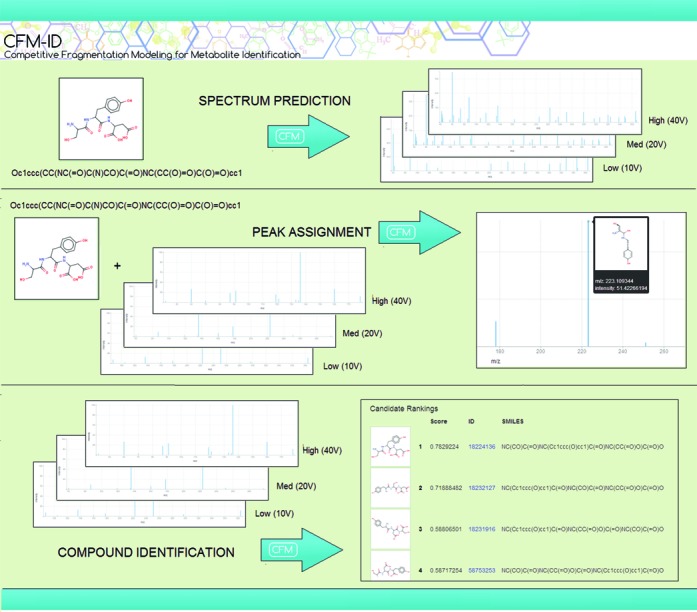
Summary of the three tasks provided by the CFM-ID web server: Spectrum Prediction, Peak Assignment and Compound Identification. Examples of possible inputs and the corresponding graphical output are shown for each.

The CFM-ID web server consists of three separate utilities as described in the following sections and summarized in Figure 1. All three make use of the same two SE-CFM models, trained using metabolite data from the Metlin database (9), as described in (8). The first model is used for all positive ionization mode computations and employs the metabolite data described in (8). This encompasses spectra for more than 1200 molecules, measured in positive ionization mode on an Agilent 6510 Q-TOF device at each of 10V (low), 20V (medium) and 40V (high) collision energies. The second model is used for all negative ionization mode computations, and has been trained using spectra for more than 800 metabolites from Metlin, also measured on an Agilent 6510 Q-TOF device at the above collision energies, but using negative ionization mode. Both models enumerate possible MS/MS fragmentations for a given molecule and assign probabilities to competing fragmentations according to the trained model parameters. The models are trained on general metabolite data, and are not specific to any particular class of compounds. Note that the peptide models described in (8) are not accessible via this server.

CFM-ID's web interface back-end was developed using the latest version of the Ruby on Rails framework. It uses MySQL and Redis for data storage, along with ChemAxon's JChem Web Services (http://www.chemaxon.com) for generating structure images. The front end uses standard HTML and Javascript technologies to input queries and display results, with the D3.js library for data visualization. When a query is submitted, it is put in a job queue, and the user is directed to a results page that updates automatically to notify the user of the running status. Jobs are run in the background using Sidekiq, which can process multiple jobs at once.

The server does not require a login to access; instead the user's results page is assigned a unique and private random ID, allowing the user to bookmark the results and access them later. These results are available for one month, after which they are removed from the server.

Runtime is dependent on the size and complexity of the user's input. For average-sized molecules (50 atoms or less), spectra prediction and peak assignment take under a minute, while larger input molecules may take a couple of minutes to process. Compound identification takes several minutes or more, depending on the number and size of the candidate compounds.

### Task 1: Spectrum Prediction

This task predicts a low (10V), medium (20V) and high (40V) collision energy MS/MS spectrum for a given chemical structure. The input chemical structure can be provided in SMILES (10) or InChI format (11), and should be a neutral molecule. A proton will then be added or removed by the program to form a [M+H]+ or [M−H]− precursor ion, according to whether the user has specified positive or negative mode ionization. The output provides the predicted spectra, and is both displayed graphically and made available in peak list format for easy download. The user can also mouse-over each of the predicted peaks to see an annotation of the fragment that is predicted to have generated that peak.

The algorithm enumerates the fragmentation possibilities in a breadth-first manner, computing the probability of each fragmentation as it goes, using the learned SE-CFM model. It then only recurs on fragments with sufficiently high probability of occurring, to produce further derivatives. Once the possibilities have been generated, the algorithm computes the predicted spectrum; it uses the marginal peak probabilities to determine the peak intensities, and presents only the top 80% of the total intensity sum, within the limits of 5 and 30 peaks, as described in a more detailed manuscript covering the CFM algorithm (8).

Common positive and negative mode adducts are also supported. The selection of an adduct other than M+H or M−H results in the addition of an extra peak to each spectrum at the precursor adduct mass. It is assumed that adducts will only be present for the precursor ion and not for any of the daughter ions. Although this is not always true, it is often the case and is an approach that has been taken elsewhere, e.g. in (12,13). We cannot use the CFM model to predict intensity values for these peaks, so they are displayed with the same intensity as the [M+H]+ (or [M−H]−) precursor ion.

### Task 2: Peak Assignment

Given one or more input spectra in peak list format and a chemical structure in SMILES or InChI format, this utility assigns putative fragment annotations to the peaks in each input MS/MS spectrum. To accomplish this, CFM-ID enumerates all possible fragments for the input structure, as described elsewhere (8), and assigns all fragments within a specified mass tolerance of each peak. Multiple fragments may be assigned to each peak, in which case they are ordered from most to least likely according to the probabilities given by the trained SE-CFM model. The input spectra are intended to be three spectra for the same compound, measured at low (10V), medium (20V) and high (40V) collision energies. However if only a subset of those spectra are available, the system will still accept the input, and annotate those spectra. Common adducts can also be assigned, however as in Task 1 these are assumed to only apply for the precursor ion and not for any of the other fragments. The other fragments are always [M+H]+ or [M−H]− according to the selected ionization mode.

A graphical depiction of each spectrum is provided in the results, with the most likely assigned fragment appearing when the user hovers his/her mouse over each peak. An example of this functionality is shown in Figure 1. For peaks with more than one possible fragment, an additional note appears stating the number of possible fragments. Clicking on the peak then displays the possibilities. A score is provided for each possibility, indicating the part of the peak intensity that is explained by that fragment. The annotations are also provided in a text file for download, with assigned fragment IDs listed after each peak in the input spectra. The fragments corresponding to those IDs are then listed underneath in SMILES format.

This utility also produces a pruned version of the deduced fragmentation graph. This comprises all fragments that form possible peak annotations, presented as a list of transitions between fragments, showing the IDs of the parent and child fragments and the neutral loss required to produce the child from the parent. This list is included in the text file, and printed on the results screen.

### Task 3: Compound Identification

This task allows users to perform putative metabolite identification for one or more input MS/MS spectra (in peak list format). The query MS/MS spectra can either be scored against a user-selected set of candidate structures, which could be input as a file from the user, or the user could select the MS/MS spectra calculated for the complete Human Metabolome Database (14) (120 000 MS/MS spectra calculated from the 40 000 compounds), or equivalently for compounds in the Kyoto Encyclopedia of Genes and Genomes (KEGG) (15). For the database searches, the user must specify the mass of the precursor peak, the tolerance on this precursor mass and the adducts to include in the search. The list of resulting candidate structures is filtered to contain only those within the specified mass range, allowing for the specified adducts.

For customized queries, the user must supply the candidate structures in a line-separated list containing reference IDs and SMILES strings. This allows the user to refine the list using any prior knowledge about the compound. A maximum of 100 candidates are allowed in the list. For longer candidate lists, it is recommended that the user run the command line utilities available at http://sourceforge.net/projects/cfm-id/ on his/her local machine.

As in Task 2, the input spectra are intended to be three MS/MS spectra for the same compound measured at low (10V), medium (20V) and high (40V) collision energies, using either positive or negative ionization mode. If only a subset of the input spectra are available, the scores will be produced using only the provided spectra. The default score is the Jaccard score averaged over collision energies as used elsewhere (8).

The mass tolerances used in peak matching can also be set, according to the known mass accuracy of the MS instrument used to measure the target spectrum.

Our system returns a list of candidates in ranked order, along with their respective scores, IDs and SMILES strings. This is displayed graphically as shown in Figure 1 and is also provided as a list for download. The user can also configure the number of top-scoring results that are reported.

## RELATED WORK

The Human Metabalome Database (14), Metlin (4) and MassBank (16) all have search functionality that query these databases for likely molecules matching an input MS/MS spectrum. The key differences between these searches and the functionality we provide (Task 3) is that they only compare the input spectrum against stored reference spectra for each compound. This restricts the possible results to the small subset of molecules whose spectra appear in these databases. In contrast, we predict the MS/MS spectra for the candidate compounds computationally, which allows us to match against any compound in a queried database, even if that database does not contain the spectrum.

A number of web servers are available that allow MS/MS spectrum searches against computationally predicted spectra for molecules from specific chemical classes, e.g. proteins (17–19) and lipids (20). However, unlike our server, these servers are not capable of predicting spectra for more general classes of metabolites.

Programs directed towards MS/MS-based identification of such metabolites include MetFrag (13) and FingerId (21), both of which have web server interfaces to their methods. Similar to our Compound Identification Utility, these servers provide utilities for putative metabolite identification using predictive algorithms. The main difference between our utility and theirs is the underlying algorithms used to perform the identification and the overall performance (see (8) and below). Both MetFrag and FingerId search for database entries within a provided mass range in public chemical databases, e.g. PubChem (22) or KEGG (15). MetFrag then uses a combinatorial enumeration of possible fragments combined with several peak scoring heuristics to determine likely candidates for a given input spectrum. FingerId uses support vector machines to predict a chemical fingerprint for a given input spectrum, and then ranks candidates by how closely they match that predicted fingerprint. In contrast, our method uses a trained, probabilistic generative model of the fragmentation process to generate predicted spectra for an input list of candidates. It then ranks the candidates in terms of how closely the predicted spectra match the provided input spectrum. Results of experimental comparisons between our method and these two methods are described in the ‘Experimental Validation’ section below.

Metlin provides a visual display of fragment annotations for the peaks in its MS/MS spectra. Unlike our Peak Assignment utility, it does not allow users to provide their own spectra for annotation. FiD (23) produces fragment annotations for a user-provided spectrum using a fragment enumeration method similar to ours, but an alternative means for determining which fragments are more likely. MetFrag provides a spectrum and annotation viewer, but the user must provide the annotations.

## EXPERIMENTAL VALIDATION

As described elsewhere (8), we performed ten-fold cross validation for the positive mode spectrum prediction and compound identification tasks, using 1491 non-peptide metabolites from Metlin.

In the spectrum prediction task, our methods predicted the peak locations with greater than 44% recall and 25% precision for non-peptide metabolites. Weighting by peak intensity, this increases to 60% recall (The sum of the intensities of the measured peaks whose corresponding predicted peak was 60% of the total sum of measured intensities) and 49% precision (The sum of the intensities of the predicted peaks whose corresponding measured peak was 49% of the total sum of predicted intensities). These values are averaged across energy levels, however the lower energy levels are generally better predicted with 77% and 68% intensity-weighted recall and precision, respectively. The testing also showed that the intensity values of matched pairs of peaks in the predicted and measured spectra had a Pearson correlation coefficient of 0.7, 0.6 and 0.45 for the low, medium and high energy spectra, respectively, indicating a positive though imperfect correlation in the intensity values.

In the compound identification tasks, we considered two methods for obtaining candidate lists. The first were produced by querying KEGG (15) for molecules within 0.5 Da of the known mass of the metabolite. In the second test case, the candidate lists were produced by querying PubChem (22) for molecules within 5 ppm of the metabolite mass. The results are summarized in Table 1. The CFM-ID performance is substantially higher than both MetFrag (13) and FingerId (21) for all data sets tested, when querying both KEGG and PubChem. When querying against KEGG, it ranked the correct metabolite first in over 75% of cases, and ranked it in the top five in more than 95% of cases. When querying against PubChem, which contains several orders of magnitude more compounds, identifying the correct compound is more difficult. The correct structure was ranked first in 10% of cases and ranked in the top 10 in over 40% of cases. Although the exact structure was not often ranked highest, the top-ranked compound was found to have the correct molecular formula in more than 88% of cases.

**Table 1.** Summary of Compound Identification results

		Querying KEGG (# cand. ≈ 22)		Querying PubChem (# cand. ≈ 1025)		
	Data Set	R = 1	R ≤ 5	R = 1	R ≤ 10	MF = 1
CFM-ID	Metlin (+)	76.5%	96.2%	10.9%	40.7%	88.9%
	MassBank	72.8%	97.5%	7.3%	46.9%	93.2%
	HMDB	23.1%	58.1%	4.1%	24.9%	88.4%
	Metlin (−)	72.1%	96.5%	13.4%	51.4%	93.8%
MetFrag	Metlin (+)	51.9%	89.9%	5.7%	30.5%	82.6%
	MassBank	48.1%	88.9%	4.7%	20.8%	85.4%
	HMDB	13.3%	43.6%	2.6%	13.4%	88.0%
	Metlin (−)	44.7%	80.7%	7.5%	28.8%	81.8%
FingerID	Metlin (+)	8.7%	36.1%	1.3%	9.3%	67.7%
	MassBank	14.8%	37.0%	0.5%	5.7%	71.9%

# cand. ≈ N : the median number of molecules in the candidate list.

R : ranking of the correct molecule in the candidate list.

MF : ranking of the correct molecular formula.

Two additional datasets were also used to explore the performance of our methods. Both were tested as described in (8) using the SE-CFM trained model from the Metlin set, as used on this server.

The first was a set of 192 metabolites from the Washington State University submission to MassBank (16). These were measured on an Agilent Q-TOF device similar to the one used to collect the Metlin data, but at a different location. As reported in (8), results for the spectrum prediction performance of this set showed only small degradation when compared to those of the cross-fold set. The intensity weighted recall was still over 60% and the intensity weighted precision was over 55%. The metabolite identification performance was fairly comparable. When querying KEGG, the correct metabolite was ranked first in over 72% of cases, and ranked in the top five in more than 97%.

The second set used 500 molecules from the Human Metabalome database (14), randomly selected from those with MS/MS data available. However, in this case the spectra were collected using a different mass spectrometer, a Quattro QqQ that has much poorer mass accuracy and a medium collision energy of 25V instead of 30V. Here the performance dropped, ranking the correct structure first in only 23.1% of cases when querying KEGG, and in the top 5 in 58.1%. For PubChem, it was only able to rank the compound in the top 10 in 24.5% of cases, however, it was still able to identify the correct molecular formula in 88.4% of cases; and still outperformed MetFrag, which ranked the correct structure in the top 10 in only 14.5% of cases.

The negative ionization model was evaluated using 10-fold cross validation on a set of 976 molecules from Metlin. These molecules were selected randomly from the non-peptide metabolites in Metlin for which negative-mode spectra were available, discarding those with a molecular weight greater than 1000 Da. In the spectrum prediction task, the recall results were lower than in the positive mode testing, scoring 23% and 50% for the non-weighted and weighted measures, respectively. However the precision values were comparable at 30% and 50% (non-weighted, weighted). The lowest energy level was again better predicted scoring 75% for both the intensity-weighted recall and precision.

In the identification task (see Table 1), the performance for the negative ionization model was comparable with that of the positive model; and again outperformed MetFrag in all tests. When querying against KEGG our method ranked the correct metabolite first in over 72% of cases and in the top 5 in over 96%.

Compound lists and full results for all tests are available in the Data section of the CFM-ID website.

## CONCLUSION

The CFM-ID web server provides a suite of tools intended to assist experimentalists in the interpretation of tandem mass spectrometry data. The server provides a user friendly web interface to the functionality in the CFM-ID software package, with graphical visualizations of molecules and mass spectra. This functionality includes spectrum prediction, peak assignment and putative compound identification. The performance has been benchmarked in cross validation testing on a large molecule set, and further validated using two additional datasets from other laboratories. It outperforms existing state-of-the-art methods, and has attained a level that could be useful to experimentalists performing metabolomics experiments.

## 

Many thanks to Dale Schuurmans, Liang Li, Stephan Beisken and Jun Peng for their useful pointers. This work was supported by the Natural Sciences and Engineering Research Council of Canada; Alberta Innovates Technology Futures; and Alberta Innovates Health Solutions and made possible by the Compute Canada Westgrid facility.

*Conflict of interest statement*. None declared.
